# Bimodal Imaging‐Visible Nanomedicine Integrating CXCR4 and VEGFa Genes Directs Synergistic Reendothelialization of Endothelial Progenitor Cells

**DOI:** 10.1002/advs.202001657

**Published:** 2020-11-09

**Authors:** Bingbo Yu, Bing Dong, Jiang He, Hui Huang, Jinsheng Huang, Yong Wang, Jianwen Liang, Jianning Zhang, Yumin Qiu, Jun Shen, Xintao Shuai, Jun Tao, Wenhao Xia

**Affiliations:** ^1^ Department of Hypertension and Vascular Disease The First Affiliated Hospital of Sun Yat‐sen University National‐Guangdong Joint Engineering Laboratory for Diagnosis and Treatment of Vascular Diseases Key Laboratory on Assisted Circulation Ministry of Health Guangzhou 510080 China; ^2^ Department of Cardiovascular The Eighth Affiliated Hospital of Sun Yat‐sen University Shenzhen 518000 China; ^3^ PCFM Lab of Ministry of Education School of Material Science and Engineering Sun Yat‐sen University Guangzhou 510275 China; ^4^ Department of Radiology Sun Yat‐sen Memorial Hospital Sun Yat‐sen University Guangzhou 510120 China

**Keywords:** bimodal imaging, cardiovascular disease, endothelial progenitor cells, nanomedicine, vascular endothelial injury repairing

## Abstract

A major challenge to treat vascular endothelial injury is the restoration of endothelium integrity in which endothelial progenitor cells (EPCs) plays a central role. Transplantation of EPCs as a promising therapeutic means is subject to two interrelated processes, homing and differentiation of EPCs in vivo, and thus a lack of either one may greatly affect the outcome of EPC‐based therapy. Herein, a polymeric nanocarrier is applied for the codelivery of CXCR4 and VEGFa genes to simultaneously promote the migration and differentiation of EPCs. Moreover, MRI T_2_ contrast agent SPION and NIR dye Cy7.5 are also loaded into the nanocarrier in order to track EPCs in vivo. Based on the synergistic effect of the two codelivered genes, an improved reendothelialization of EPCs is achieved in a rat carotid denuded model. The results show the potential of this bimodal imaging‐visible nanomedicine to improve the performance of EPCs in repairing arterial injury, which may push forward the stem cell‐based therapy of cardiovascular disease.

## Graphical abstract

A bimodal imaging‐visible nanodrug integrating chemokine receptor 4 (CXCR4) and vascular endothelial growth factor (VEGFa) genes is prepared to effectively codeliver two genes to endothelial progenitor cells (EPCs) and to track the EPCs therapy in vivo. Owing to the synergistic effect of the two genes, an improved reendothelialization of EPCs is achieved in a rat carotid denuded model.

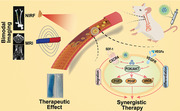

## 1 Introduction

Cardiovascular disease (CVD) is the leading cause of death worldwide, leading to more than 17.5 million deaths per year.^[^ ^1^, ^2^ ^]^ Admittedly, loss of the normal endothelial structure and function underlies the initiation and development of CVD, which makes the maintenance of endothelial integrity an important strategy to reduce the high incidence and mortality of CVD.^[^ ^3^, ^4^, ^5^ ^]^ Accumulating evidences indicate that the circulating endothelial progenitor cells (EPCs) provide an important endogenous repair mechanism in maintaining the integrity of vascular endothelium after arterial injury, and transplantation of EPCs is considered to be an effective strategy to protect the structural and functional stabilities of vascular endothelium.^[^ ^6^, ^7^, ^8^ ^]^ However, the ineffective migration to vascular lesion and weakened proliferation/differentiation of EPCs to mature endothelial cells (ECs) existed in the presence of CVD risk factors such as hypertension, ageing, diabetes, which greatly limit the beneficial effects of EPCs on the repair of vascular endothelial injury.^[^ ^9^, ^10^, ^11^ ^]^ Therefore, development of a potent approach to improve the performance of EPCs in vivo may enhance the EPC‐based endothelial repair to reduce the CVD occurrence.

It is well known that the vascular endothelial growth factor (VEGFa), a dominant inducer to the growth of blood vessels, is closely related to the survival, proliferation and differentiation of EPCs.^[^ ^12^, ^13^, ^14^ ^]^ So far, many attempts have been made to direct the EPCs differentiation into ECs in a hope to enhance reendothelialization. For instance, Keun‐Hong Park et al. used nanogel to mediate gene transfection and upregulated the VEGFa expression of EPCs, which significantly enhanced differentiation of EPCs into ECs and thus promoted neovascularization in an ischemia model.^[^ ^15^ ^]^ Yet, other critical processes such as EPCs homing should be considered as well in the EPCs‐based therapies. Obviously, the success of stem cell transplantation therapy for vessel reendothelialization depends on both the differentiation of EPCs into ECs and an efficient EPCs homing to sites of endothelial injury. Moreover, increasing evidences show that chemokine receptor 4 (CXCR4), the receptor of chemokine stromal cell‐derived factor‐1 (SDF‐1), is a pivotal factor in regulating the EPCs homing to and retention at the sites of injured artery.^[^ ^16^, ^17^ ^]^ Our previous studies have revealed that an upregulation of CXCR4 signaling pathway through exercise, fluid shear stress and drugs can boost up the migration and homing capacity of EPCs and also improve the endothelial function in the elderly and hypertensive patients.^[^ ^18^, ^19^, ^20^ ^]^ Therefore, a combined gene therapy via simultaneously upregulating the CXCR4 and VEGFa expressions of EPCs may enhance the homing and differentiation potential of EPCs to improve their revascularization potential, which depends on an efficient codelivery of the two genes. To our knowledge, a combined gene therapy of CXCR4 and VEGFa to direct the reendothelialization of EPCs remains unexplored yet. In addition, a potent noninvasive imaging tool to monitor the in vivo homing process is vital for a rational design of such multifunctional gene delivery systems.

Recent studies have demonstrated that nonviral carriers including cationic lipids and polymers may effectively deliver nucleic acids, e.g., plasmid DNA (pDNA) and siRNA, into various cells like cancer cells^[^ ^21^, ^22^ ^]^ and stem cells.^[^ ^23^, ^24^ ^]^ We have also demonstrated highly efficient gene regulation by using polymeric carriers to mediate pDNA and siRNA delivery.^[^ ^25^, ^26^ ^]^ A directed differentiation of neural stem cells was achieved through gene delivery with polymeric vector. More excitingly, incorporating the magnetic resonance imaging (MRI) contrast agents such as superparamagnetic iron oxide nanocrystals (SPION) may endow the vectors with MRI visibility, which offers a great opportunity to track the in vivo fate of the transplanted stem cells.^[^ ^26^, ^27^ ^]^ Among available medical imaging modalities, MRI possesses the advantages of high spatial resolution, allowing 3D visualization of anatomic structures.^[^ ^28^, ^29^, ^30^ ^]^ Nevertheless, the imaging sensitivity of MRI is much lower than that of the optical imaging. Thus, a combination of the two imaging modalities is likely a feasible approach for better imaging which integrates high spatial resolution and high sensitivity.^[^ ^31^, ^32^ ^]^ In particular, a combination with the near‐infrared (NIR) imaging is highly attractive because of the superior tissue penetration and minimal background autofluorescence of NIR lights in the range of 650–900 nm.^[^ ^33^ ^]^ Therefore, polymeric carriers with MRI/NIR bimodal imaging functions may provide desirable platforms for codelivery of CXCR4 and VEGFa into EPCs in vitro and then for monitoring of the transplanted cells in vivo.

**Scheme 1:**
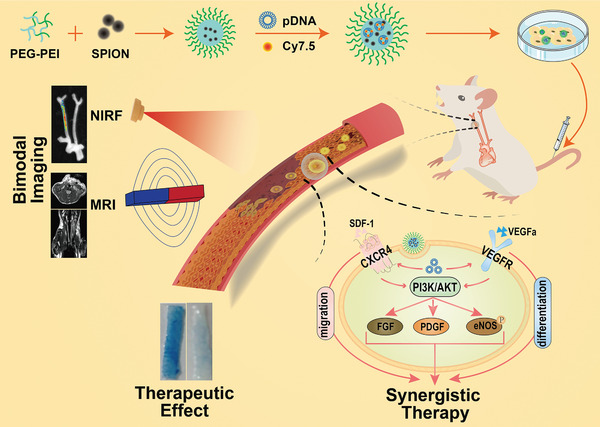
Schematic illustrations of bimodal imaging‐visible nanomedicine (PEG‐PEI) integrating CXCR4 and VEGFa genes directs synergistic reendothelialization of endothelial progenitor cells.

Herein, a bimodal imaging‐visible nanodrug integrating CXCR4 and VEGFa genes was prepared using cationic copolymer PEG‐PEI as a nonviral vector to load pDNA as well as MRI contrast agent SPION and NIR dye Cy7.5, with the dual aims to effectively codeliver two genes into EPCs and to track the EPCs therapy in vivo. Hopefully, when the multifunctional nanodrug entered EPCs, the two genes would be released, transcripted and translated to promote both the EPCs homing capacity and directed differentiation, thereby resulting in a synergistic effect to enhance reendothelialization (**Scheme** **1**). In vitro and in vivo experiments were carried out to explore the potential of this theranostic nanodrug to improve the performance of EPCs in repairing arterial injury, which expectantly may push forward the stem cell‐based therapy of CVDs.

## 2 Results and Discussion

### 2.1 Preparation and Characterization of MRI‐Visible pDNA‐Complexed Nanoplex

**Figure 1:**
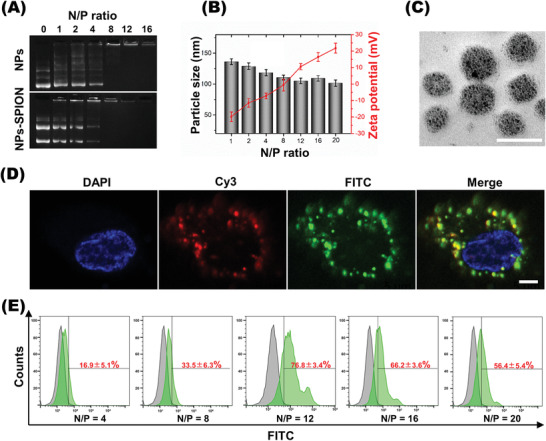
Characterization of cDNA plasmid‐complexed and SPION‐loaded nanoplex. A) Electrophoretic mobility of cDNA plasmid in agarose gel after complexing with mPEG‐PEI (NPs) and mPEG‐PEI/SPION (NPs‐SPION) at various N/P ratios. B) Particle size and zeta potential of nanoplexes at various N/P ratios. Data are shown as mean ± SD, *n* = 3. C) Morphology of nanoplex prepared at N/P 12 as measured by transmission electron microscope (TEM). Scale bar: 200 nm. D) Fluorescence microscopic images revealed pDNA (green) and nanocarrier (red) inside EPCs transfected with pDNA/NPs‐SPION. Scale bar: 2 µm. E) N/P ratio‐dependent transfection efficiency quantified using flow cytometry assay (*n* = 5).

The cationic copolymer PEG‐PEI was synthesized by conjugating CDI‐activated PEG to PEI as described in Figure S1B,C (Supporting Information). SPION, a T_2_ contrast agent for MRI, was used in consideration of its biocompatibility and high MRI T_2_ sensitivity. Then, the MRI‐visible nanocarrier PEG‐PEI‐SPION (NPs‐SPION) was prepared by ligand exchange method,^[^ ^34^ ^]^ through which the PEG‐PEI replaced the hydrophobic coating of oleic acid on the surface of SPION measuring 6 nm. The SPION weight percentage of the polymer‐coated nanoparticles was determined to be 55% by Fe atomic absorption assay. Then, NPs‐SPION was employed to complex both CXCR4 pDNA and VEGFa pDNA, and the ability of NPs‐SPION to complex pDNA was evaluated using agarose gel electrophoresis. It is well known that the negative charge of pDNA would be neutralized after complete complexation with cationic nanocarrier, leading to a loss of its motility under electric field.^[^ ^35^ ^]^ As shown in **Figure** **1**A, pDNA was fully complexed at N/P ratios above 12. As excess carrier materials may potentially impose unexpected side effects in vivo,^[^ ^36^ ^]^ the nanodrug which was a nanoplex formed at N/P = 12 was chosen for further experiments. In addition, the cationic nanoparticles without SPION showed similar complexation ability, suggesting that the SPION loading had no obvious effect on the pDNA complexation. This was reasonable in consideration of the high positive charge of PEG‐PEI‐SPION nanoparticles.

Dynamic light scattering (DLS) measurements were performed to study the complexation between NPs‐SPION and pDNA at various N/P ratios. As shown in Figure 1B, when the N/P ratio increased, the size of nanoplex, NPs‐SPION/pDNA, slightly decreased, whereas the zeta potential of NPs‐SPION obviously increased, which was due to the increase of uncomplexed cationic amino groups on the surface of nanoparticles at higher N/P ratios. The nanoplexes with weak positive charge are reported to possess low cytotoxicity and tend to be easily endocytosed by cells,^[^ ^37^, ^38^ ^]^ thus improving the gene transfection efficiency. Therefore, the nanodrug formed at N/P 12 seemed appropriate for the pDNA delivery in consideration of its zeta potential (10.6 ± 1.9 mV).

Under transmission electron microscopy (TEM) observation, NPs‐SPION/pDNA formed at N/P 12 showed spherical morphology and uniform particle size distribution around 78.7 ± 9.5 nm in diameter. The mean hydrodynamic diameter of NPs‐SPION/pDNA measured by light scattering was 105.1 ± 4.5 nm, featuring a narrow polydispersity index (PDI) (Figure 1C; Figure S1D, Supporting Information). Laser scanning confocal microscopy (LSCM) was used to evaluate the cell uptake and intracellular distribution of the fluorescently labeled pDNA. To locate the nanocarrier, the nuclei were stained blue with DAPI. As shown in Figure 1D, the cells incubated for 6 h showed very strong green fluorescence from the FITC‐labeled pDNA and red fluorescence from the Cy3‐labeled PEG‐PEI, and the two kinds of scattered fluorescent dots overlapped in the cytoplasm. These results strongly indicated that the nanocarrier mediated efficient pDNA delivery into EPCs via an endocytosis pathway. To determine the optimized N/P ratio for efficient transfection, EPCs were incubated with NPs‐SPION/vector at various N/P ratios. Flow cytometry analysis revealed that EPCs incubated with nanoplex of N/P 12 showed the most prominent cellular uptake of pDNA, which reached a high transfection efficiency up to 76.8 ± 3.4% (Figure 1E).

**Figure 2:**
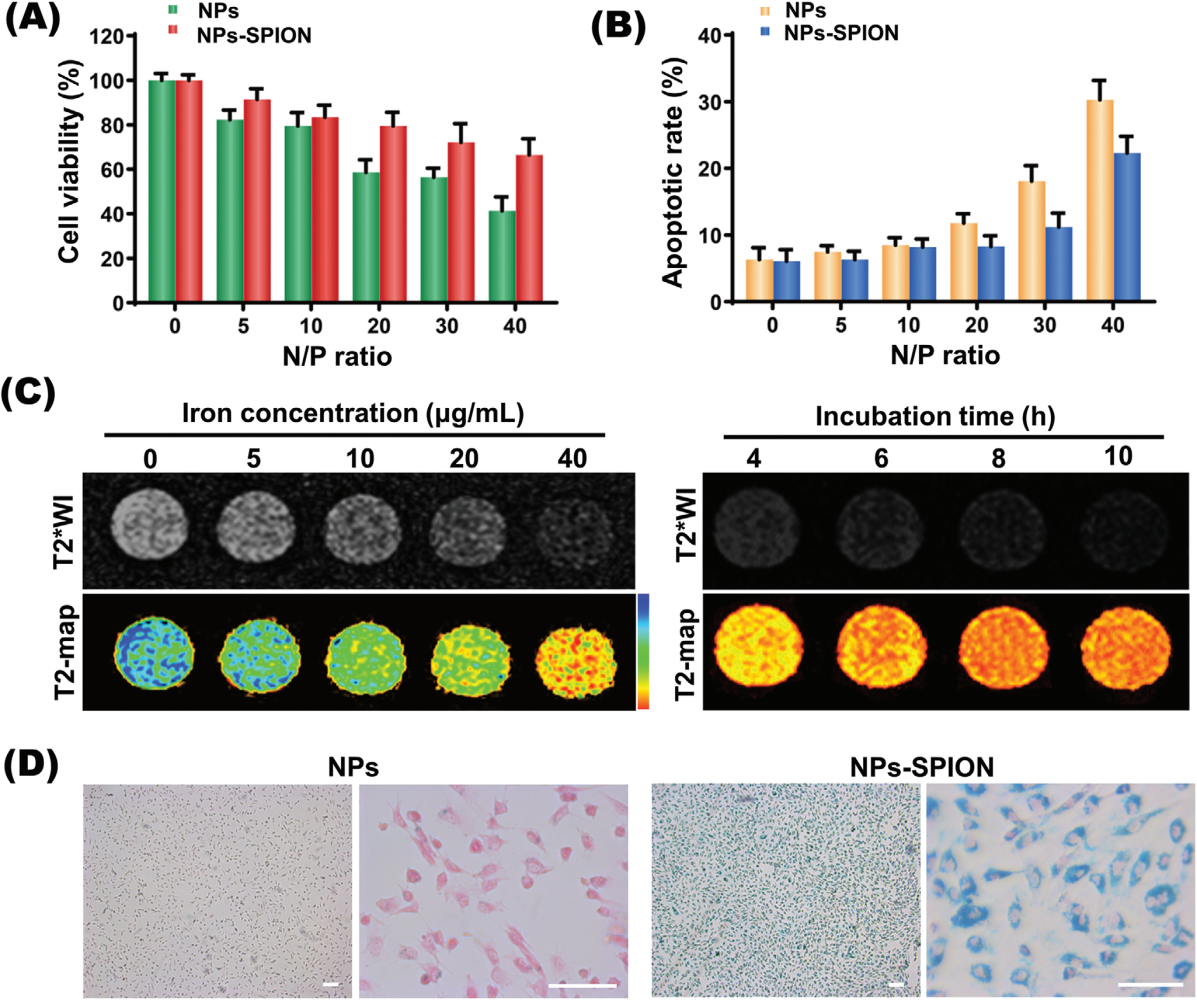
Cytotoxicity and cellular uptake. A,B) Cell viabilities and apoptosis rates detected by CCK8 and by flow cytometry at various N/P ratios in EPCs transfected with NPs and NPs‐SPION. Data are shown as mean ± SD, *n* = 3. C) T_2_‐weighted imaging (T_2_WI) and pseudocolored imaging of EPCs after incubation with NPs‐SPION at various Fe concentrations and time. D) Prussian staining (blue) microscopic images showed abundant iron particles inside EPCs transfected with NPs‐SPION in contrast to EPCs transfected with SPION‐free (NPs) control. Scale bar: 100 µm.

The CCK8 assay and Annexin‐V/PI cytometry analysis were performed to evaluate the EPC viability and apoptosis, respectively. As shown in the CCK8 assay (**Figure** **2**A,B), both the SPION‐free and SPION‐encapsulated nanoplexes showed higher cytotoxicity at higher N/P ratios, and encapsulation of SPION obviously reduced the cytotoxicity of nanoplex likely due to the decrease in surface charge (Figure S1E, Supporting Information). Notably, the cells incubated with NPs‐SPION formed at N/P 12 showed a high viability above 88.5% (100 µg mL^−1^ of NPs‐SPION), which indicated a low cytotoxicity suitable for gene transfection study.

Encapsulation of SPION into the nanocarrier enabled MRI monitoring of gene delivery into EPCs which were incubated with nanoplex (N/P = 12) integrating CXCR4 and VEGFa (pDNA‐CXCR4/VEGFa). As shown in Figure 2C, the T_2_‐weighted signal intensity of MRI was affected by both the dose and incubation time. In other words, the T_2_‐weighted signal intensity was obviously weakened along with an increase in the iron concentration or incubation time. Consistently, Prussian blue staining showed that cells incubation with nanocarrier led to abundant intracellular iron distribution (Figure 2D). In consideration that cell incubation at high iron levels or for prolonged time may decrease cell viability, the Fe concentration of 40 µg mL^−1^ and 8 h incubation were adopted as the properly optimized conditions for EPC transfection in the following experiments.

### 2.2 Effect of Theranostic Nanodrug on Migration and Differentiation of EPCs

**Figure 3:**
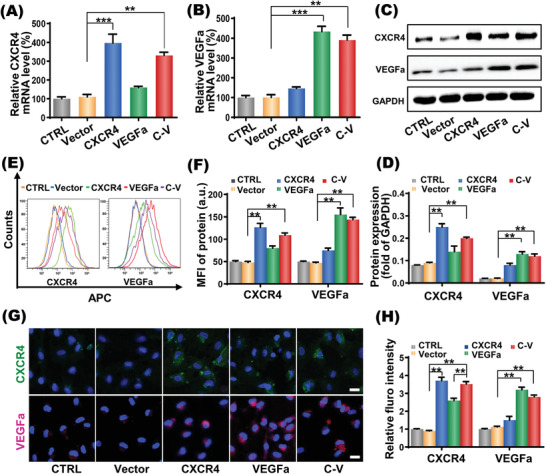
In vitro gene expression regulation of EPCs. A,B) CXCR4 and VEGFa mRNA expression in EPCs transfected with Vector/NPs‐SPION(Vector), CXCR4/NPs‐SPION(CXCR4), VEGFa/NPs‐SPION(VEGFa), CXCR4‐VEGFa/NPs‐SPION(C‐V) and cells without treatment (CTRL). C) Representative photographs and D) quantification analyses of CXCR4 and VEGFa protein expressions in transfected EPCs assessed by Western blotting. E) Representative photographs and F) quantification analyses of CXCR4 and VEGFa protein expressions in transfected EPCs assessed by flow cytometry. G) Representative photographs and H) quantification analyses of CXCR4 and VEGFa protein expressions in transfected EPCs evaluated by laser confocal scanning microscopy (LCSM). Scale bar: 10 µm. Data are shown as mean ± SD, *n* = 5. **P < 0.01, ***P < 0.001.

CXCR4 gene plays an important role in regulating stem cell survival and migration via interaction with ligand SDF‐1, while VEGFa is believed to promote differentiation and proliferation of EPCs for angiogenesis. Therefore, codelivery of the two genes to EPCs may not only improve the homing capacity but also promote the proliferation and directed differentiation of EPCs, thus synergistically enhancing EPC‐based stem cell therapy. To determine the transcription and protein expression of delivered genes, quantitative polymerase chain reaction (qPCR) and Western blot (WB) analyses were conducted at 3 d after cell transfection. Transfection with nanoplex carrying pDNA‐CXCR4/VEGFa (C‐V group) simultaneously increased the mRNA levels of CXCR4 and VEGFa by 330.7 ± 16.65% and 390.7 ± 25.01%, respectively (**Figure** **3**A,B). Western blot analysis obtained consistent results. That is, nanoplex carrying two genes increased the protein expression levels of CXCR4 and VEGFa by 2.8 times and 5.3 times respectively (Figure 3C,D), whereas the nanoplexes carrying one gene, i.e., NPs‐SPION/CXCR4 or NPs‐SPION/VEGFa, only showed comparable efficiency to increase the protein expression of a single gene. In addition, flow cytometry and enzyme‐linked immunosorbent assay (ELISA) analysis also confirmed the synergistic effect of NPs‐SPION/C‐V on expressions of CXCR4 and VEGFa (Figure 3E,F, Figure S3, Supporting Information), and LSCM image of EPCs after immunofluorescence staining directly showed the upregulated expressions of the intracellular CXCR4 and VEGFa proteins (Figure 3G).

**Figure 4:**
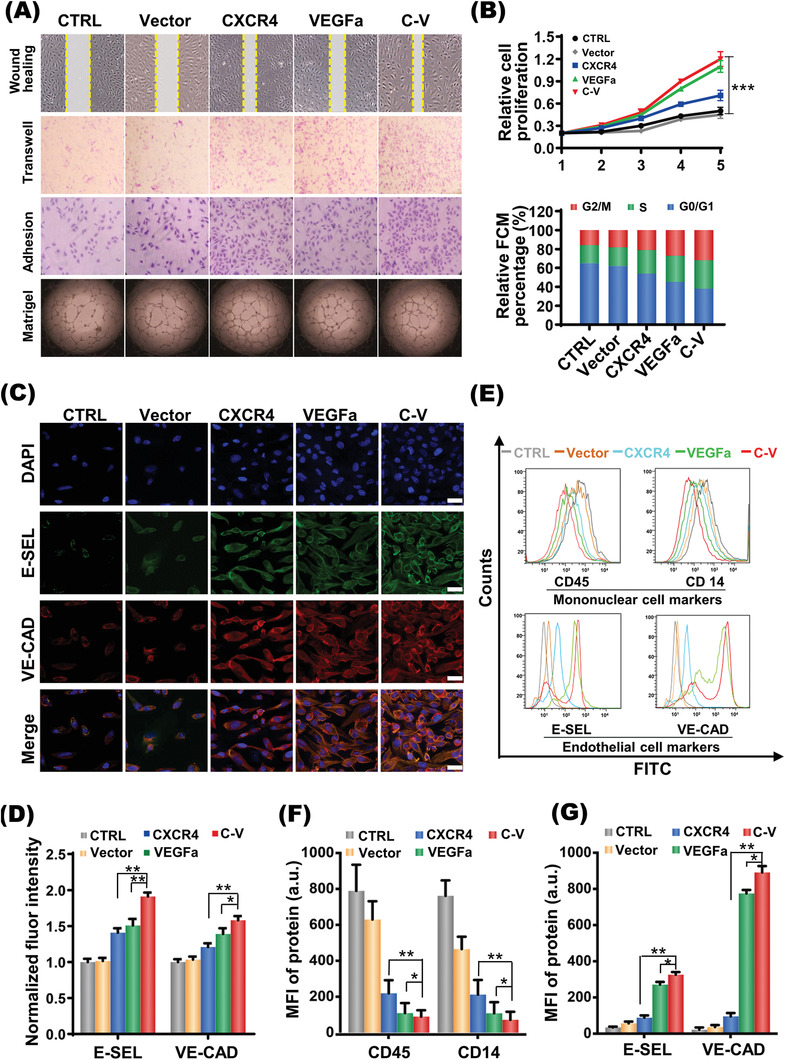
Migration and differentiation of EPCs enhanced by codelivery of CXCR4 and VEGFa genes. A) Migration (wound healing and transwell), adhesion, tube formation (Matrigel) of EPCs in different groups (five groups: CTRL, Vector, CXCR4, VEGFa, C‐V). B) EPCs proliferation (upper panel) and cell cycle (lower panel) of different groups. C,D) Immunofluorescent staining (C, Red: VE‐CAD; Green: E‐SEL) for endothelial cell markers and semiquantification D) on EPCs after 2 week culture in different groups. Scale bar: 10 µm. E–G) Histograms of flow cytometric analysis of cell markers on EPCs after 14 d culture in different groups. Data as mean ± SD, *n* = 5. *P < 0.05, **P < 0.01, ***P < 0.001. Abbreviations: CTRL, cells without treatment; Vector, EPCs transfected with Vector/NPs‐SPION; CXCR4, EPCs transfected with CXCR4/NPs‐SPION; VEGFa, EPCs transfected with VEGFa/NPs‐SPION; C‐V, EPCs transfected with CXCR4‐VEGFa/NPs‐SPION; FCM, flow cytometry; VE‐CAD, vascular Endothelial‐Cadherin; E‐SEL, E‐ selectin.

As shown in **Figure** **4**A, the wound‐healing and transwell assays showed that the EPCs with CXCR4 upregulation (CXCR4 group) more significantly enhanced the EPCs migration than the cells without treatment (CTRL group) or with vacant plasmid transfection (Vector group), which was consistent with previous reports that the CXCR4 gene plays an important role in EPC migration.^[^ ^19^, ^39^ ^]^ Moreover, the VEGFa transfection group (VEGFa group) also showed enhanced wound healing and meanwhile increased cell number crossing the transwell porous membrane in comparison with the CTRL and Vector groups, which was due to the increased amount of cells in consideration that VEGFa may promote not only the differentiation of EPCs into ECs but also the proliferation of both EPCs and ECs.^[^ ^40^, ^41^ ^]^ As a result, EPCs with both CXCR4 upregulation and VEGFa upregulation (C‐V group) even more effectively enhanced wound healing and increased the number of migrated cells than EPCs with either CXCR4 upregulation or VEGFa upregulation, which also implied that the two genes would likely show a synergistic effect on the repair of endothelial injury. According to the immunofluorescence staining for the endothelial lineage markers such as VE‐cadherin and E‐selectin (Figure 4C,D),^[^ ^42^, ^43^ ^]^ elevated expression levels of VE‐cadherin and E‐selectin were shown in the VEGFa group, which provided more direct evidence for the promoted differentiation of EPCs. Although the CXCR4 transfection facilitated somewhat the ECs differentiation of EPCs likely via the PI3k‐AKT pathway,^[^ ^44^, ^45^, ^46^ ^]^ the VEGFa transfection showed much stronger effect on the ECs differentiation of EPCs. Moreover, the CXCR4 and VEGFa cotransfection (C‐V group) showed a more powerful effect to promote the differentiation of EPCs than the VEGFa transfection group.

The flow cytometry analysis further showed that, at 2 weeks after transfection, the cells lost the mononuclear cell markers such as CD14 and CD45 but gained the endothelial cell markers such as VEGFR, Tie2 E‐selectin especially in the VEGFa and C—V groups (Figure 4E,F).

The effects of CXCR4 and VEGFa expressions on proliferation, cell cycle, adhesion and angiogenesis of EPCs were investigated. Notably, EPCs in the C‐V group demonstrated the mostly enhanced cell proliferation and the most cells in the S/G2phases of cell cycle (Figure 4B). Moreover, cells in the VEGFa group and the C‐V group showed very similar behaviors due to the dominant effect of VEGFa expression on EPCs proliferation. These cells showed obviously higher level of proliferation than the cells in the CXCR4 groups. In addition, the adhesion and tube formation assays indicated that EPCs in the C‐V group had the greatest potential to adhere to injured endothelium and to form neovasculature (Figure 4A; Figure S4, Supporting Information). Taken together, the above results strongly demonstrated that the two gene transfection (C‐V group) represents a highly effective means to strengthen the ability of EPCs to repair the endothelial injury through simultaneously improving their differentiation, proliferation, migration, and adhesion.

**Figure 5:**
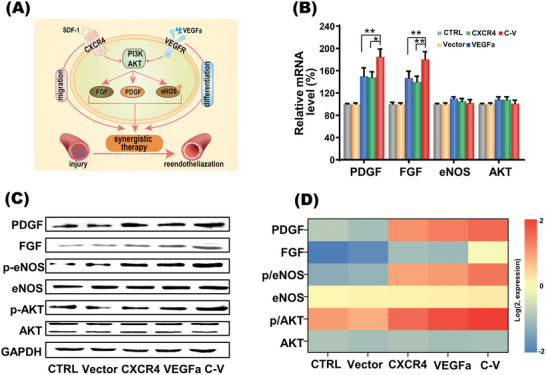
The synergy of CXCR4 and VEGFa genes codelivered into EPCs. A) Schematic illustration of the synergistic reendothelialization of combination therapy with CXCR4 and VEGFa genes transfected in EPCs. B) The mRNA expressions of related molecular targets in EPCs receiving CXCR4 and VEGFa codelivery quantified by qPCR analysis. Data are shown as mean ± SD, *n* = 3. *P < 0.05, **P < 0.01. C) The protein expressions of related molecular targets in EPCs receiving CXCR4 and VEGFa codelivery analyzed by Western blot. D) Density quantification of protein expression of related molecular targets in EPCs receiving CXCR4 and VEGFa codelivery. Abbreviations: PDGF, platelet derived growth factor; FGF, fibroblast growth factor; eNOS, endothelial nitric oxide synthase; AKT, protein kinase B; GAPDH, glyceraldehyde‐3‐phosphate dehydrogenase.

Except for their major roles in the proliferation, differentiation and migration of EPCs, previous studies also found that CXCR4 and VEGFa share the PI3k‐AKT signaling pathway (**Figure** **5**A), which may explain the enhanced adhesion and angiogenesis of the two genes transfection group. As indicated by the WB assay (Figure 5B–D), codelivery of CXCR4 and VEGFa resulted in the activation of PI3k‐AKT signaling pathway, which led to the upregulated expressions of phosphorylated endothelial nitric oxide synthase (eNOS) and growth factors including fibroblast growth factor (FGF) and platelet derived growth factor (PDGF). These molecules are widely considered essential for angiogenesis and revascularization. Therefore, the enhanced revascularization via CXCR4 and VEGFa codelivery in vitro may result from the synergistic effect of eNOS, FGF and PDGF regulations by PI3k‐AKT. These encouraging results have driven us to further test the effect of codelivered CXCR4 and VEGFa on revascularization in animal model.

### 2.3 Biological Functions of Transplanted EPCs In Vivo

**Figure 6:**
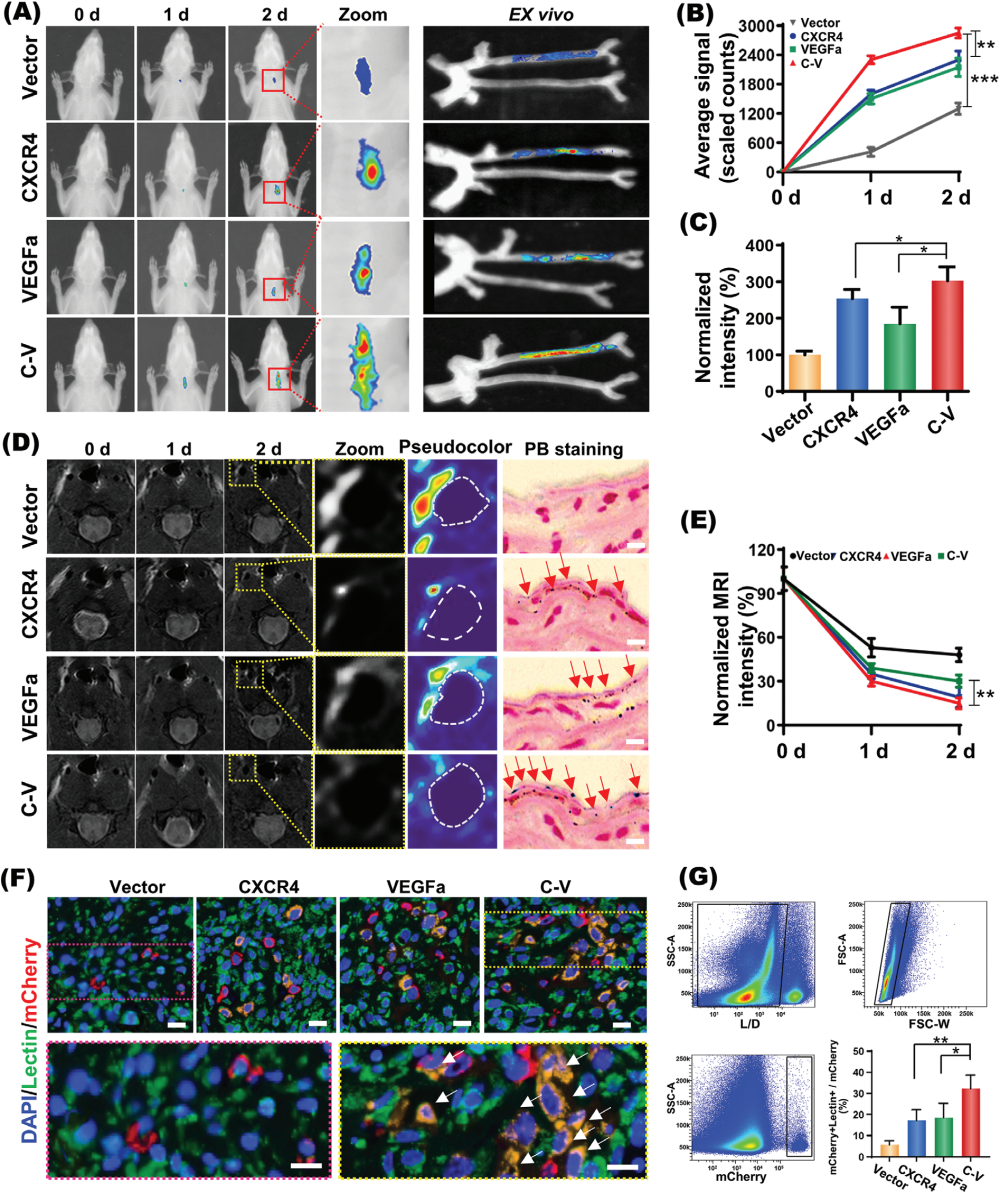
In vivo effects of codelivered CXCR4 and VEGFa genes on migration and differentiation of EPCs. A) in vivo NIR optical imaging in different time points (days: 0, 1 and 2) and ex vivo fluorescence imaging of the separated carotid at day 2 after tail vein injection of transfected‐EPCs labeled with Cy7.5 into the right carotid injury nude rat. B) Average fluorescence signal quantification of the areas of interest shown in (A). C) Normalized ex vivo fluorescence intensity of the carotid separated at day 2 as shown in the right panel of (A). D) Coronal T_2_‐weighted MR images of the injured carotid artery of nude rat after injecting transfected‐EPCs at different time points (days: 0, 1, and 2). The right panel showed Prussian blue staining (PB staining) of the injured right carotid sections of different groups at day 2. The red arrows mark the SPION (blue). Scale bar: 10 µm. E) Normalized MRI T_2_ signal intensity of the injured carotid artery endothelium of nude rat after injecting transfected‐EPCs. F) Fluorescent assays indicating EPCs persistently expressing the red fluorescence protein mCherry (mEPCs) in the injured carotid sub‐endothelium at 3 d after transplantation. Scale bar: 20 µm. G) Quantitative assessment of EPC differentiation into endothelial cells (double positive for mCherry and Lectin) by flow cytometry. Data are shown as mean ± SD, *n* = 6. **P < 0.01,***P < 0.001.

Admittedly, multimodal imaging is a powerful technique for monitoring the survival and biodistribution of implanted cells in vivo.^[^ ^44^, ^47^ ^]^ Considering that EPCs need to exert a therapeutic effect in lesion site,^[^ ^48^ ^]^ it is important to visualize the migration and incorporation of implanted EPCs to the sites with endothelial injury in response to the chemotactic signals. EPCs were incubated with nanoparticles conjugated with Cy7.5, a hydrophobic near infrared fluorescent agent, and then in vivo fluorescence imaging was performed at 0 d (immediately after injection), 1 d, 2 d (*N* = 6 per time point) after the cell transplantation. As shown in **Figure** **6**A,B, the fluorescence intensity of the right carotid artery region gradually increased within 2 d after injection of EPCs labeled with NPs‐SPION, indicating the incorporation of EPCs to the injured site. Although the two groups with single gene transfection showed better effect than the control group, the C‐V group displayed the highest fluorescence signal intensity at all time points, which indicated the best effect to enhance EPCs migration and homing via the CXCR4 and VEGFa codelivery. Determination of the fluorescence signals from the isolated carotid arteries obtained results in line with that obtained in vivo (Figure 6A–C). Moreover, microscopic observation of the injured right carotid common artery (RCCA) with CD31 immunofluorescence staining (green fluorescence) further confirmed that, at 2 d after tail vein injection, EPCs incubated with Cy3‐NPs‐SPION/C‐V (red fluorescence) homed most effectively in the injured RCCA (Figure S5, Supporting Information). In comparison, EPCs incubated withCy3‐NPs‐SPION/CXCR4 or Cy3‐NPs‐SPION/VEGFa only showed moderate homing effect at the injured RCCA.

As an effective toll in detecting vascular injury^[^ ^49^ ^]^ and arteriosclerosis^[^ ^25^ ^]^ in animal models,^[^ ^50^ ^]^ magnetic resonance imaging (MRI) was utilized to monitor the in vivo temporal‐spatial distribution and therapeutic effect of EPCs in animal model with endothelium injury. SPION, which is a type of negative MRI contrast agent, will weaken the MRI T_2_ signals of tissues where they are situated. As shown in Figure 6D,E, the intravenously injected EPCs showed gradual homing to the injured endothelium in all groups, leading to a gradual decline of MRI T_2_ signal intensity there. Apparently, EPCs incubated with NPs‐SPION/C‐V (NPs‐SPION/C‐V‐EPCs) showed the best homing to the injured endothelium. For example, at 2 d after injecting EPCs via tail vein, rat receiving NPs‐SPION/Vector‐EPCs showed hyperintense MRI T_2_ signal at the injured carotid artery. In comparison, although rat receiving NPs‐SPION/VEGFa‐EPCs or NPs‐SPION/CXCR4‐EPCs exhibited obvious decline in the MRI T_2_ intensity, rat receiving NPs‐SPION/C‐V‐EPCs presented the most hypointense MRI T_2_ signal at the injured carotid artery (Figure 6D,E). Prussian blue staining assay provided direct evidence that the SPION‐incorporated nanoparticles were delivered to the injured carotid tissues via EPCs homing. As shown in the right panel of Figure 6D, the most abundant blue stains were observed in the carotid artery of rat receiving NPs‐SPION/C‐V‐EPCs, confirming once again the best EPCs homing to the injured endothelium.

The fate of implanted cells was evaluated by fluorescent assays, in which EPCs persistently expressing the red fluorescence protein mCherry (mEPCs) were employed to enable the detection of ECs differentiated from EPCs (Figure S6, Supporting Information). As shown in Figure 6F, mEPCs regardless of transfection with Vector, CXCR4, VEGFa, C‐V were found in the injured carotid subendothelium at 3 d after transplantation. Moreover, transfection with CXCR4 or VEGFa may enhance the endothelial differentiation of EPCs in vivo. More excitingly the NPs‐SPION/C‐V transfection exhibited the best effect according to the highest amount of neovascular endothelial cells at the subendothelium of injured carotid artery. These results suggested the synergistic effect of CXCR4 and VEGFa on EPC differentiation. Quantitative assessment of EPC differentiation by flow cytometry obtained consistent results (Figure 6G). In the injured endothelium border zone, the percentage of ECs differentiated from mEPCs transfected with NPs‐SPION/C‐V(32.3 ± 6.31%) was significantly higher than those of ECs differentiated from mEPCs transfected with NPs‐SPION/CXCR4 (17.2 ± 5.08%) or NPs‐SPION/VEGFa (18.7 ± 6.64%). Collectively, these results demonstrated that the codelivered CXCR4 and VEGFa synergistically enhanced the EPC differentiation in vivo, which is critical for the EPC‐mediated reendothelialization.

### 2.4 Therapeutic Effect of Transplanted EPCs In Vivo

**Figure 7:**
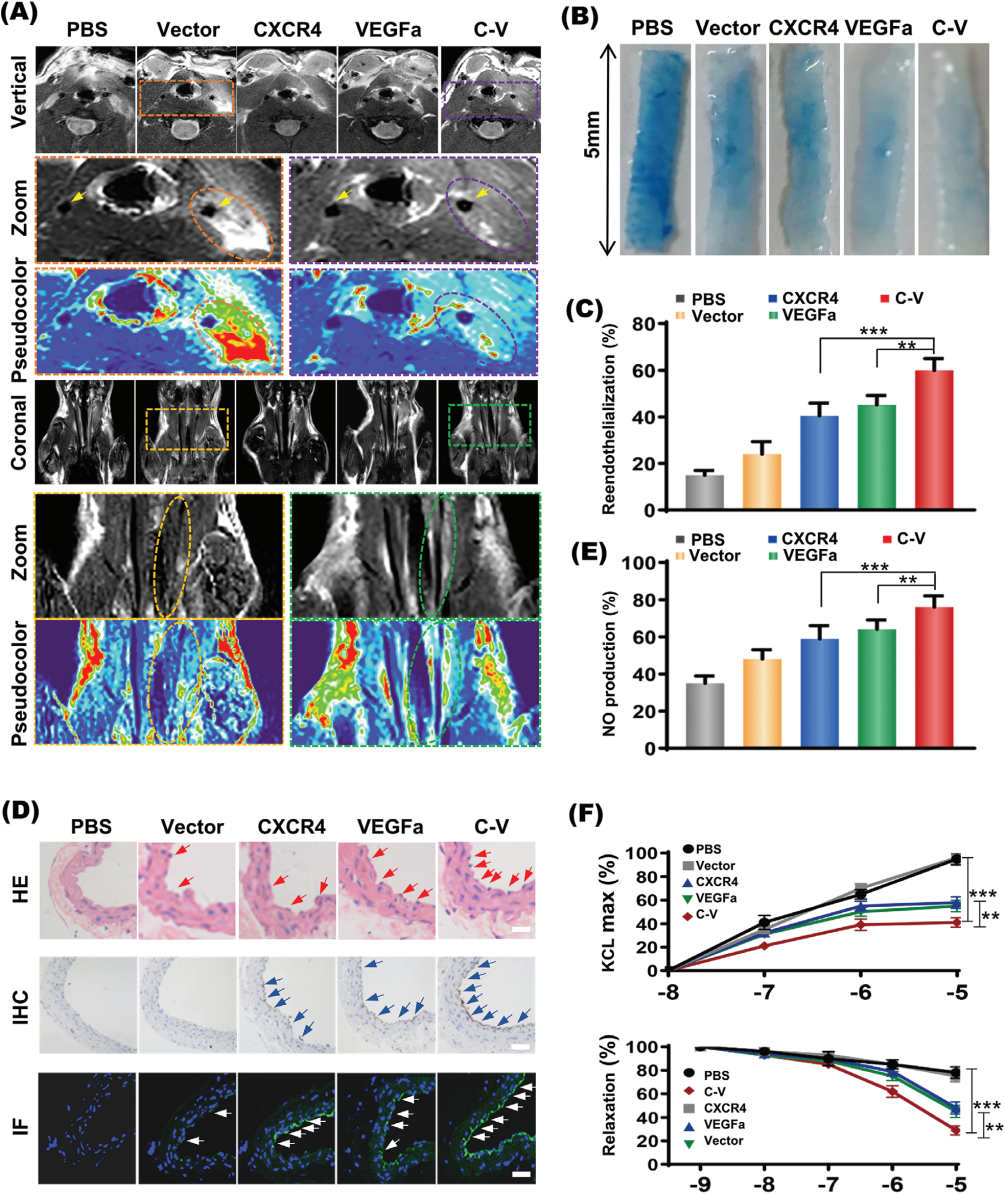
The therapeutic effect of transplanted EPCs in vivo. A) Representative T_2_WI serial MR imaging for coronary (upper three panel) and transverse (lower three panel) views of carotid sections at day 3 of different groups. The amplified and pseudocolored image of the dotted area is presented below. B,C) Reendothelialization areas on the injured carotid arteries of different groups by Evans blue staining. Denudated‐endothelium area was stained in blue; reendothelialization area was in white and its quantification (C). D) Representative micrographs of hematoxylin‐eosin (HE) staining of EC (upper panel), immunohistochemistry (IHC) staining of CD31 (middle panel) and immunofluorescence (IF) staining of *α*V*β*3 (lower panel) in injured carotid of nude rats. Scale bar: 5 µm. E) Nitric oxide (NO) released from endothelial cells of injured carotid from different groups. F) The expression of phenylephrine induced vasoconstriction and the expression of vasorelaxation of carotid artery to acetylcholine stimulation assesses by Aortic vasorelaxation assay. Data are shown as mean ± SD, *n* = 6. **P < 0.01, ***P < 0.001.

MRI scan was performed at 3 d after transplantation to evaluate the therapeutic effects of EPCs transfected with CXCR4 and/or VEGFa on the carotid artery regeneration. As shown in **Figure** **7**A, compared to the left carotid common artery without injury (yellow arrows) showing hypointense MRI T_2_ signals, hyperintense MRI T_2_ signals were detected in the injured carotid well of the PBS and vector groups due to the inflammatory exudation. Obviously, the implanted EPCs with CXCR4 and/or VEGFa transfections effectively weakened the MRI T_2_ signals of the injured carotid well due to the reduced inflammatory exudation, among which the C‐V transfection showed the most prominent effect. In addition, clear therapeutic effect of nanodrug was also verified based on the pathological changes of vascular intima in animals receiving different treatments. That is, the PBS and vector groups showed coarse intima, whereas the three therapeutic groups, in particular the C‐V group, displayed smoother intima, which were confirmed by the histological HE staining (Figure 7D). Coronal MRI showing vascular longitudinal section obtained the same results, which demonstrated less inflammatory exudation and pathological changes of vascular intima upon gene transfection of EPCs. Histological examinations showed no pathological change in liver and kidney and the serum biochemical marks (BUN, CR, ALT, and AST) of rats were all within the normal ranges before and after EPCs transplantation up to 14 d (Figure S7A,B, Supporting Information). Furthermore, we tested the effect of SPION on the lifespan of differentiated endothelial cells by CCK‐8 assay. Difference was not observed between the cells containing SPION and cells containing no SPION (Figure S7C, Supporting Information). These results imply that SPION will not affect the lifespan of differentiated endothelial cells in vivo.

The therapeutic effects of implanted EPCs with CXCR4 and/or VEGFa transfection on the structural and functional recovery of arterial endothelium were finally assessed. The blood perfusion of the rat carotid in different groups was monitored with the laser doppler flowmetry before and after EPCs transplantation, which were shown in Figure S8A,B (Supporting Information). In comparison with the CTRL, CXCR4 and VEGFa groups, the C‐V group showed significantly enhanced carotid blood perfusion recovery. The in vivo endothelial repair capacity of EPCs, defined as non‐Evans blue staining area/total denuded area, was significantly improved in the C‐V group (Figure 7B,C). In other words, compared to the PBS injection, all the implanted EPCs showed obvious effect to reduce the injured areas stainable by Evans blue staining, and the implanted EPCs receiving C‐V transfection showed the most potent efficacy among the three transfection groups. The C‐V group showed the most remarkedly preserved regeneration of the endothelium. Based on the Immunofluorescence staining (Figure 7D), the CXCR4 and VEGFa groups showed significantly higher expression level of *α*V*β*3, a surface marker of neonatal endothelial cells than the PBS and vector groups, and the C‐V group displayed the highest *α*V*β*3 expression among all groups. In addition, the five groups showed the identical patterns for the protein expressions of CD31, EC surface markers. These results suggested the therapeutic functions of EPCs through the enhanced EPC recruitment and differentiation into ECs due to the synergistic effect of CXCR4 and VEGFa.

Further, the nitric oxide release and vasorelaxation assays were carried out to test the protective roles of CXCR4 and VEGFa in the carotid relaxation functions. In comparison with the PBS group, the CXCR4, VEGFa and C‐V groups showed significantly enhanced NO release from the carotid endothelium (Figure 7E). More importantly, this performance was most significantly punctuated in the C‐V group. The PE‐induced vasoconstriction was significantly decreased in the C‐V group, as compared with the CXCR4 and VEGFa groups. Furthermore, the Ach‐induced vasorelaxation was opposite to that of the PE‐induced vasoconstriction (Figure 7F). These results implied that the injury‐associated vascular problems, i.e., the impaired endothelial function and vasorelaxation, could be comprehensively improved via the synergistic therapeutic effect of the codelivered CXCR4 and VEGFa.

Finally, we observed the changes of genetic profile of the host endothelial cells and the injected engineered EPCs by examining the mRNA levels of endothelialization‐related biological molecules (CXCR4, VEGFa, VWF, VCAM‐1, FGF, PDGF, and eNOS) and apoptosis‐related genes (Bcl‐2 and Bax) of host endothelial cells and injected engineered EPCs (Figure S9, Supporting Information). The results showed that transplantation of the engineered EPCs upregulated the expression of endothelialization‐related biological molecules and downregulated apoptosis‐related genes of endothelial cells in the injured site. Notably, our results are in line with previous reports that EPCs can promote the repair of injured endothelial not only through a direct differentiation into mature endothelial cells but also through a paracrine effect.^[^ ^51^, ^52^ ^]^ In contrast, the genetic profile of the engineered EPCs after transplantation was fairly stable except for the moderately elevated expressions of endothelium biomarkers (VWF, VCAM‐1) due to directed differentiation.

## 3 Conclusion

Aiming to enhance the migration and differentiation of EPCs for highly effective revascularization, we developed bimodal imaging‐visible nanocarrier for efficient codelivery of CXCR4 and VEGFa genes into EPCs. The MRI/NIR imaging visible nanodrug showed advantages to provide noninvasive imaging of EPCs migration and homing. The upregulated expressions of the CXCR4 and VEGFa genes in EPCs resulted in the remarkably enhanced endothelial differentiation of exogenous EPCs, which promoted functional recovery in wire induced endothelial injury. This type of theranostic nanodrug that can visualize and control the EPCs behaviors in vivo provides opportunities to develop potent cell‐based therapies for CVD.

## 4 Experimental Section

##### Plasmids

The pcDNA‐CXCR4, pcDNA‐VEGFA, and null pcDNA plasmids were described in the previous report^[^ ^53^ ^]^ and used in the present study (Figure S1A, Supporting Information). The plasmid DNAs were amplified using *Escherichia coli* and purified according to the manufacturer's instructions of EndoFree Plasmid Giga Kits (QIAGEN, CA, USA). The purified pDNAs were kept in endotoxin‐free TE buffer at a concentration of 2.4 mg mL^−1^ prior to use.

##### Preparation and Characterization of Gene‐Loaded Nanoparticle

The SPION‐PEG‐PEI/pDNA nanoparticle was prepared as follows. First, mPEG‐PEI was synthesized by two step reactions.^[^ ^54^, ^55^ ^]^ The mPEG‐CDI was obtained by activation of monomethoxy (polyethylene glycol) (mPEG‐OH, *M*_n_ = 2 kDa) with *N,N*′‐carbonyldiimidazole (CDI). The mPEG‐CDI was conjugated to branched polyethylenimine (PEI, *M*_n_ = 25 kDa) to obtained PEG‐PEI. Second, the PEG‐PEI was coated to oil‐soluble SPION with an average diameter of 6 nm by ligand exchange reaction, obtaining SPION‐PEG‐PEI (Figure S1B,C, Supporting Information).^[^ ^56^ ^]^ Third, the SPION‐PEG‐PEI was complexed with pDNA through electrostatic interactions in water to obtain SPION‐PEG‐PEI/pDNA.

##### Determination of DNA Complexation

In order to assess the pDNA condensation ability of the delivery agents, gel electrophoresis was performed on a Bio‐Rad Sub‐Cell electrophoresis cell (Bio‐Rad Laboratories, Inc., USA). Complex formation was induced at various N/P ratios (calculated as the number of nitrogen atoms in delivery agents over that of the phosphate groups in pDNA) from 0 to 80 in a final volume of 6* agarose gel loading dye mixture (i.e., 10 mL). pDNA (4 µg) and the appropriate amount of delivery agent were dissolved separately in a neutral solution containing 0.9% sodium chloride. The two solutions were mixed by vigorous pipetting, loaded onto the 0.9% agarose gels with ethidium bromide (0.1 mg mL^−1^), and ran with Tris–acetate (TAE) buffer at 100 V for 40 min. DNA band shifts were revealed by irradiation with UV light.

##### Zeta Potential and Size Measurements

The sizes and zeta potentials of SPION/PEG‐PEI/plasmid particles were determined by DLS measurements. Zeta potential measurement of nanoparticles was carried out in the standard capillary electrophoresis cells, using a ZetaPlus instrument (Brookhaven, NY, USA) at a position of 45° angle at 25 °C. The average values plus standard deviations were based on the data of five runs. Nanoparticle size was determined on the same instrument at 25 °C. Scattering light was detected at 90° angle, and the sizes given are the means of five runs plus standard deviations.

##### Cell Culture and Identification

Peripheral blood mononuclear cells were isolated by ficoll density gradient centrifugation as described previously.^[^ ^18^, ^19^ ^]^ EPCs were cultured and characterized by following previously described.^[^ ^18^, ^19^, ^20^ ^]^ Briefly, after 4 d culture, nonadherent cells were removed by thoroughly washing with endothelial cell basal medium‐2 (EBM‐2) (Clonetics). Medium was changed every 3 d, and the cultured cells were used for following experiments.

To confirm the EPCs phenotype, the attached EC‐liked cells were incubated with 1,1′‐dioctadecyl‐3,3,3′,3′‐tetramethylindo‐carbocyanineperchlorate‐labeled acetylated LDL (DiI‐acLDL; Molecular Probes, Eugene, OR, USA) at 37 °C for 1 h. The cells were then fixed with 4% paraformaldehyde for 30 min at 37 °C, and incubated with fluorescein isothiocyanate (FITC)‐labeled lectin (Sigma‐Aldrich, St. Louis, MO, USA) for 4 h at 37 °C. After being stained, the samples were observed with a phase‐contrast fluorescent microscope. Cells demonstrating double‐positive fluorescence were identified as differentiating EPCs. After 2 weeks culture, marker proteins of cultured EPCs were examined by flow cytometry analysis using CD31 (BD), kinase‐insert domain receptor (KDR) (R&D) and vWF (R&D) mouse antihuman antibody (Figure S2A,B, Supporting Information). Based on the isolation and cultivation protocol,^[^ ^18^, ^20^ ^]^ the adherent mononuclear cells were identified as EPCs.

##### Optimized Transfection Condition

The vector backbone plasmid LeGo‐iC2 (mCherry) was purchased from Addgene (Cambridge, MA). To produce lentiviral vectors, the backbone plasmids were transfected with envelope (pMD2.G) and packaging (psPAX2) plasmids into 293FT cells; 2 d later, virus‐containing supernatants were collected and concentrated by ultracentrifugation. To infect EPCs, the lentivirus was applied to the cells with polybrene (8 µg mL^−1^). The medium was replaced on the following day, and the cells were cultured for an additional 3 d before fluorescence‐activated cell sorter sorting for the transduced cells based on mCherry expression. 5 × 10^5^ EPCs were seeded in each cell of 96‐well plates containing 200 µL EBM‐2 medium to explore the optimized transfection conditions. For the best SPION‐NPs concentration in transfection, each well was added with various amounts of SPION‐NPs (N/P 12) to reach the final iron concentration of 0, 5, 10, 20, 40 µg mL^−1^, respectively. Then, the cells were incubated for 6 h under 37 °C and 5% CO_2_. For the ideal transfection time, cells were incubated with SPION‐NPs at 40 µg mL^−1^, when the concentration of cDNA was 120 × 10^−9^ m, for various time of 4, 6, 8,10 h under 37 °C and 5% CO_2_. In the above two tests, untreated cells served as control. After transfection, the medium was discarded and the cells were washed with PBS to eliminate residual nanoparticle. Finally, the cellular uptake of the SPION‐NPs was measured by in vitro MRI.

##### In Vitro MRI Scan

Nanoparticles were diluted with various amounts of PBS to reach various final iron concentrations and cells were resuspended in 200 µL 1% agarose solution, and then in vitro MRI assays were performed on a clinical 3.0 T MR unit (Intera; Philips Medical Systems, Best, The Netherlands) with an 8‐channel SENSE knee coil. Fast spin echo (FSE) T_1_‐weighted images and T_2_WI and fast field echo (FFE) T_2_*‐weighted images (T_2_*WI) were obtained to observe the intracellular uptake of SPION mediated by SPION‐CP. T_2_ maps were obtained using single‐section multi‐spin echo sequences to acquired T_2_ relaxation times. The detailed acquisition parameters were described previously.^[^ ^57^ ^]^ All experiments were conducted in triplicate.

##### Safety of Nanomedicine Transfection on EPCs

To assess the safety of nanomedicine transfection on EPCs, cell viability and apoptosis were determined. Cell viability was determined by cell counting kit‐8 (CCK‐8) assay according to the manufacturer's instruction. Briefly, after nanomedicine transfection, 1 × 10^4^ EPCs were cultured in flat‐bottom 96‐well plates for another 1, 2, or 3 d. After 10 µL of CCK‐8 solution (Kumamoto, Japan) was added to incubate the cells for another 4 h, the absorbance at 450 nm was recorded on a microplate reader (SpectraMaxM5; Molecular Devices, CA, USA). Cell apoptosis was assessed by Annexin V/propidium iodide double staining method and analyzed using a flow cytometry 24 h after transfection. Untreated NSCs served as control. All experiments were conducted in triplicate.

To determine the transfection efficiency, cells were incubated with FITC labeled SPION‐NPs and free‐labeled Vector (120 × 10^−9^ m) respectively under various N/P conditions after 8 h. The quantitative analysis of cellular uptake of FITC labeled Vector was performed by using flow cytometry. For flow cytometry, transfected EPCs were dissociated into single cells and sorted on flow cytometry (CytoExpert, Beckmann Coulter). The percentage of FITC positive cells was counted to show the transfection efficiency. Untreated cells were used as control. All these experiments were performed in triplicate. Data were analyzed using the BD Flow Jo 10.5 software (BD Bioscience USA).

##### Internalization and Distribution of Polyplexes into EPCs

To enable CLSM observation of the cells after incubation, the delivery agent PEG‐PEI‐SPION was labeled with FITC and pDNA was labeled with CY3 according to the manufacturer's protocols. The EPCs were seeded in six‐well plates at a density of 1 × 10^6^ cells per well and cultured with the EGM‐2 medium. pDNA and the delivery agent PEG‐PEI‐SPION were mixed at N/P of 12 for 30 min at room temperature to allow the formation of the polyplex of pDNA and delivery agent. Then the mixture was added into six‐well plates and cultured 60 min. Then EPCs were fixed with 4% paraformaldehyde for 20 min and incubated with DAPI for 5 min at room temperature to counterstain nuclei. The fluorescence signals of cells were detected on a laser scanning confocal microscope (LSCM710; Carl Zeiss, Jena, German). Cells incubated with free ‐labeled Vector (120 × 10^−9^ m) were used as control.

Intracellular distribution of SPION was detected by Prussian blue staining. Briefly transfected cells were fixed and incubated with Prussian blue solution containing 10% hydrochloride and 10% potassium ferrocyanide (II) trihydrate for 30 min at 37 °C, and then counterstained with nuclear fast red. Cells incubated with free Vector (120 × 10^−9^ m) were used as control. For in vivo study, full‐thickness histological sections of carotid were collected at 3 d after injection for analysis of tumor Fe accumulation using Prussian blue staining.

##### RNA Isolation and Real‐Time Polymerase Chain Reaction

EPCs were transfected with SPION/PEG‐PEI/plasmid (C‐V, CXCR4, VEGFa, Vector) under optimized transfection conditions. RT‐qPCR was performed at 48 h after transfection according to the method. Total RNA was extracted with the high pure RNA isolation kit (TransGen). RT‐qPCR was carried out by the routine three‐step method. cDNA products were amplified by the following primer pair for target gene coding sequence. The CXCR4 coding sequence: forward, 5‐TCTTCCTGCCCACCATCTACTC‐3 and reverse 5‐GTAGATGACATGGACTGCCTTGC‐3. VEGFa sequence: forward 5′‐ACTTTCTGCTGTCTTGGGTG‐3′ and reverse, 5′‐CTGCATGGTGATGTTGGACT‐3′ Gene expression was analyzed by using the iQ SYBR Green Supermix and iQ5 Real‐Time PCR detection system (Bio‐Rad). The mRNA level of GAPDH gene was measured in each sample as an internal normalization standard.

##### Western Blot Analysis

Total EPCs protein were extracted and quantified by protein extraction reagent (Merck) and bicinchoninic acid protein assay kit (Thermo Fisher) separately. Protein extracts were subjected to SDS‐PAGE, transferred to polyvinylidene fluoride membranes (Roche). The following antibodies were used: rabbit anti‐CXCR4 antibody (1:500; ABCAM, USA), rabbit anti‐actin antibody (1:2000; Cell Signaling Technology), rabbit anti‐VEGFa antibody rabbit (1:500; Santa Cruz, USA), rabbit anti pan protein kinase B (1:1000, Abcam), p‐Akt (1:2000, Ser473; Abcam), rabbit anti human PDGF B (1:2000, Abcam), goat anti human FGF 23 (1:800, Abcam), mouse anti human eNOS (1:500 Abcam), p‐eNOS (1:500 Ser1177; Abcam), and rabbit anti‐GADPH antibody (1:3000; Cell Signaling Technology). Proteins were visualized with HRP‐conjugated anti‐rabbit or anti goat IgG (1:2000; Cell Signaling Technology), followed by use of the ECL chemiluminescence system (Thermo).

##### Flow Cytometry for Expression of Transfected Genes

For cell surface staining, samples were stained with APC‐labeled mouse anti human CXCR4 (1:100, BD) at 4 °C for 30 min. Then cells were washed twice with PBS and resuspended in 1% formaldehyde buffer before analysis. For intracellular staining, cells were first incubated with brefeldin for 6 h and then fixed and permeabilized using a Cell Permeabilization Kit (BD Biosciences, San Jose, CA, USA) before incubating with Alexa Fluor labeled mouse anti human VEGF (NO). Flow cytometry was performed as above.

##### ELISA Assay of Conditioned Media

Cell culture media were saved from EPCs in different groups. VEGFa levels in the cell culture conditioned medium were measured using Human VEGF DuoSet ELISA kits (R&D Systems, Minneapolis, MN, USA) with antibodies mainly specific for VEGFa according to the manufacturer's instructions.

##### In Vitro Migration Function of EPCs

The migration of EPCs was tested with two different assays. For transwell assay (Corning, New York, NY), EPCs were digested and resuspended in serum‐free EGM‐2 medium (EBM‐2 with supplements), and subsequently, 2 × 10^4^ cells were loaded into the upper chambers. The lower chamber was filled with EGM‐2 medium and SDF‐1 (100 ng mL^−1^, PeproTech). After incubation at 37 °C for 6 h, EPCs were stained with crystal violet and counted in three random fields (magnification, × 200) in each well. For Wound‐Healing Assay, 2 × 10^5^ EPCs were plated in six‐well culture dish. A wound was created by manually scraping the cell monolayer with a p20 pipet tip, followed by wash once and supplemented with 1 mL EBM‐2 with or without SDF‐1 (100 ng mL^−1^, PeproTech). After 16 h incubation at 37 °C, transmigrated cells were observed under an optical microscope by independent investigators blinded to treatment groups randomly.

##### In Vitro Differential Function of EPCs

To determine the effect of VEGFa‐CXCR4 codelivery on differentiation of EPCs in vitro, 5 × 10^5^ EPCs transfected with SPION/PEG‐PEI/plasmid (C‐V, CXCR4, VEGFa and Vector) were induced to differentiate after 14 d of induction. The differentiation of EPCs was characterized by immunostaining for E‐selectin and VE‐cadherin expression, as described above. The endothelial differentiation of cells was observed using a fluorescence microscope (BX63; Olympus, Tokyo, Japan). The quantification of endothelial differentiation capacity was analyzed using Image J software. The percent of differentiation was calculated as the total number of nuclei present divided by the number of VE‐cadherin or E‐selectin positive cells. Untreated cells were used as control. The quantification of endothelial differentiated from EPCs was also validated using flow cytometry. Briefly, cells were collected by centrifugation, resuspended in 100 µL of FACS buffer containing 1 µg of the indicated antibody, dispensed in a minimum of 10^5^ cells per sample, gently mixed, and incubated on ice for 30 min. Flow cytometry (CytoExpert, Beckmann Coulter) and data analysis software Flow Jo software version 10.5 (TreeStar, Inc, Ashland, OR) were used.

##### In Vitro Adhesion and Tube Formation Function of EPCs

EPC adhesion assay was performed as described. Dishes were coated with fibronectin (10 µg mL^−1^). EPCs (2 × 10^4^) of different groups in each well of a 24‐well plate were stimulated with SDF‐1 (100 ng mL^−1^) for 6 h at 37 °C. Nonattached cells were removed with PBS, and adherent EPCs were fixed with 4% paraformaldehyde and stained with 0.3% crystal violet. The adherent EPCs were counted by independent investigators blinded to treatment groups randomly. For tube formation assay, a growth factor‐reduced Matrigel (Corning) was warmed up at 4 °C overnight. After completely thawed, 60 µL of Matrigel was plated to 96‐well plates at the same level to distribute evenly and incubated for 1 h at 37 °C. EPCs (2 × 10^4^) were resuspended with EBM‐2 and loaded on the top of the Matrigel. Each conditional group contained three wells. Following incubation at 37 °C for 12 h, each well was imaged directly under a microscope, and an average of tubules was counted from three to five random fields.

##### In Vitro Proliferation and Cell Cycle of EPCs

Cell proliferation and cell cycle assays were performed as described above. In brief, for cell proliferation, 10^3^ cells were seeded in a 96‐well plate on day 0 with the treatment. Absorbances at 490 nm were measured using CellTiter 96 AQueous One Solution Cell Proliferation Assay kit (Promega) on day 2, day 4, and day 6 to measure the cellular proliferation. For cell cycle, cells were washed with PBS twice and resuspended in 1% formaldehyde buffer. After incubation with propidium for 30 min, samples were assessed with CytoExpert, Beckmann Coulter.

##### Wire‐Injury of Rat Carotid Artery

Based on our previous work,^[^ ^58^ ^]^ wild‐type male mu/mu Nude RAT (Beijing Vital River Laboratory Animal Technology Company, Beijing, China), and aged 8–10 weeks were used to allow to inject EPCs. Animals were anesthetized with an intraperitoneal injection of pentobarbital sodium (50 mg kg^−1^). Surgery was carried out using a dissecting microscope. Through a middle line neck incision on the ventral side, the right common carotid artery, including bifurcation, was exposed. A bulldog clamp was placed around right common carotid artery proximal to the aortic arch for temporary control of blood flow, and 6‐0 suture was placed around external carotid artery. An incision hole was made in the right external carotid artery, then a flexible wire (0.58 mm) was introduced into common carotid artery and passed three times toward and forth with rotation to denude endothelium. Right external carotid artery was tied off proximal to the incision hole after wire was removed. The skin incision was closed with surgical sutures. Three days after transplantation, endothelial regeneration was evaluated as reendothelialization area of the denudated endothelial zone by staining with 50 µL of solution containing 5% Evans blue dye via tail vein injection. The dye‐stained area was quantified. Image analysis was performed.

EPCs (25 × 10^5^ cells) of different groups resuspended in 500 µL of prewarmed PBS (37 °C) were transplanted after 3 h of wire‐injury via tail vein injection with a 28‐gauge needle, and the same volume of PBS was injected as placebo control. All experimental protocols followed the Guide for the Care and Use of Laboratory Animals published by the US National Institutes of Health (National Institutes of Health Publication No. 85‐23, revised 1996) and the Animal Care and Use Committees of Sun Yat‐sen University (Guangzhou, China).

The common carotid artery blood flow was performed using a VisualSonics Vevo 3100 ultrasound biomicroscope (VisualSonics Inc, Toronto, ON, Canada). B‐mode data and pulsed‐wave Doppler data were acquired at 40 MHz, with a 30 framesper second (fps) frame rate. The diameter of common carotid artery and mean velocity were detected by using pulsed‐wave Doppler ultrasound. Results were processed using VisualSonics analysis software.

Serum samples of rats in different groups were collected for serum biochemical tests. Alanine aminotransferase (ALT) and aspartate aminotransferase (AST) were used to evaluate liver injury while creatinine (CR) and blood urea nitrogen (BUN) were utilized to assess renal function.

##### In Vivo and Ex Vivo Fluorescence Imaging

Cy7.5 instead of Cy3 was loaded into PEG‐PEI micelle for fluorescence imaging in the same way as that for preparation of Cy3‐loaded micelles. Before imaging study, the rats of different groups were anesthetized by intraperitoneal injection of pentobarbital sodium (50 mg kg^−1^). Optical imaging was performed using a small animal in vivo fluorescence imaging system (Carestream, USA) with emission at 720 nm and excitation at 790 nm after the mice were injected with the polyplex solutions. The fluorescence images were captured at different time points. The fluorescence intensity was quantified using Carestream MI software. Ex vivo imaging study of carotid was performed as well under the same image.

##### In Vivo MRI

After anesthetized, animals were placed in the prone position. MRI was performed on a clinical 3.0‐T MR scanner (Intera; Philips Medical Systems) with a 60 mm × 60 mm four channel phased array rat coil. MRI scan was performed at 1, 2, 3 d after tail vein injection of EPCs to detect the distribution and migration of transplanted cells and reendothelialization. Axial and coronal brain images were obtained by using axial and coronal FSE T_2_WI (TR = 800 ms, TE = 60 ms, and NSA = 2) and FFE T_2_*WI (TR = 500 ms, TE = 18 ms, flip angel = 20°, and NSA = 3). Other acquisition parameters were FOV = 60 × 60 mm^2^, matrix = 256 × 256, and section thickness = 1.0 mm. All MRI images were blindly evaluated by one observer experienced in reading rat MRI images. The entire volume with hypointense signal on T_2_*WI MRI was evaluated to indicate the distribution and migration of transplanted cells.

##### Aortic Vasorelaxation and Nitric Oxide Release

The rat carotid segments in each animal were cleaned and cut into slices of 2 mm in length to evaluate the contractile and relaxant response according to our previous reports. In brief, the aortic rings were carefully mounted on an isometric force transducer with a tension of 1.8 g and placed in an organ chamber filled with Krebs solution maintained at pH 7.4 and bubbled with 95% O_2_ and 5% CO_2_. After an equilibration of 40 min, 1 µmol L^−1^ of phenylephrine (PE) was added to the organ chamber for the assessment of contractile activity, and then 30 µm L^−1^ of acetylcholine (ACh) was added to assess the endothelial integrity. After washing and re‐equilibration for 30 min, a cumulative PE dose (from 1 nmol L^−1^ to 1 µmol L^−1^) was added to the organ chamber to obtain a concentration‐dependent contractile curve. Then sodium nitroprusside (30 µmol L^−1^) was added to the organ chamber to obtain a relaxant response. After washing and re‐equilibration for 20 min, 30 µmol L^−1^ of ACh was added into the organ chamber, followed by 1 µmol L^−1^ of PE to evaluate the endothelium‐dependent vasorelaxant response. The PE (1 µmol L^−1^)‐induced vasocontractile response was assessed again in the presence of L‐NAME (100 µmol L^−1^) pretreatment for 30 min. All data were acquired and analyzed using the Panlab system (Panlab Harvard Apparatus, Barcelona, Spain). Vascular basal NO release was calculated as the percentage difference between PE‐induced vasocontractile response in the presence and absence of L‐NAME.

##### In Vivo Differentiation and Reendothelialization of Immunohistochemical (IHC) and Immunofluorescent (IF) Staining

To assess in vivo synergistic effect of CXCR4 and VEGFa on EPC differentiation, carotid artery was harvested and fixed in 4% paraformaldehyde for 4 h and then snap‐frozen. IF was performed using fluorescent anti‐BS lectin 1 (Vector Laboratories, Inc) and DAPI. Three sections per carotid and 6 fields per section were examined. Immunoreactive signals were observed by fluorescence microscope (BX63; Olympus, Tokyo, Japan). Carotid arteries were collected and digested for 1 h at 37 °C in an enzymatic mix (10 mg/ml Collagenase type II (C6885, Sigma Aldrich) and 1 mg mL^‐1^ Elastase (LS002292, Worthington Biochemistry) to single cell for flow cytometry analysis. To explore the influence of the engineered EPCs transplantation on host endothelial cells in vivo, digested aortas were mechanically disrupted and immediately processed for injected EPCs (mCherry expression) by FACS. The mRNA levels of endothelialization‐related biological molecules (CXCR4, VEGFa, VWF, VCAM‐1, FGF, PDGF and eNOS) and apoptosis‐related genes (Bcl‐2 and Bax) were assessed with qPCR quantification in the sorted EPCs and ECs.

To assess in vivo therapeutic effect, analysis was performed on carotid sections obtained from rats. IHC staining for CD31, IF staining for *α*v*β*3 were conducted according to the method as before mentioned. Briefly, carotids were harvested and coronal sections of 8 µm thickness were prepared. rehydrated paraffin sections were first treated with 3% H_2_O_2_ for 10 min and incubated with Immuno‐Block reagent (BioSB, Santa Barbara, CA, USA) for 30 min at room temperature. Sections were then incubated with primary antibodies specifically against CD31 (1/100, Abcam), *α*v*β*3 (1/200, Abcam). Three sections specimen from each rat were analyzed. For quantification, three randomly selected HPFs were analyzed in each section. An observer, experienced in molecular biology, blindly evaluated all histologic and immunohistochemical images to quantify in cell or tissue specimens. All surgical procedures and pathohistological analyses were performed by investigators blinded to treatment assignments.

##### Statistical Analysis

The sample sizes were carefully chosen for each experiment on the basis of pilot experiment examinations and sufficient statistic powers. For all animal studies, at least six animals per group were used to ensure the adequate power. All the results were compared by using standard two tailed Student's *t*‐test or one‐way analysis of variance. All the sample sizes were proved to be appropriate for assumption of normal distribution and variance was similar between the compared groups. The statistical values of *P* < 0.05 were considered statistically significant. The values of mean determinants are presented as mean ± SD.

## Conflict of Interest

The authors declare no conflict of interest.

## Supporting information

Supporting Information
