# Supplementary material for: Bimodal Imaging‐Visible Nanomedicine Integrating CXCR4 and VEGFa Genes Directs Synergistic Reendothelialization of Endothelial Progenitor Cells
Source: Adv Sci (Weinh). 2020 Nov 9;7(24):2001657. doi: 10.1002/advs.202001657 (PMC7740091; doi:10.1002/advs.202001657)
Supplement: Supplementary file 1 — Supporting Information [file ADVS-7-2001657-s001.pdf]

© 2020 Wiley-VCH GmbH

ADVANCED SCIENCE
Open Access

Supporting Information

for *Adv. Sci.*, DOI: 10.1002/advs.202001657

Bimodal Imaging-Visible Nanomedicine Integrating CXCR4 and VEGFa Genes Directs Synergistic Reendothelialization of Endothelial Progenitor Cells

Bingbo Yu, Bing Dong, Jiang He, Hui Huang, Jinsheng Huang, Yong Wang, Jianwen Liang, Jianning Zhang, Yumin Qiu, Jun Shen, Xintao Shuai, Jun Tao,* and Wenhao Xia**

Supporting Information

Bimodal Imaging-visible Nanomedicine Integrating CXCR4 and VEGFa Genes Directs Synergistic Reendothelialization of Endothelial Progenitor Cells

Bingbo Yu, Bing Dong, Jiang He, Hui Huang, Jinsheng Huang, Yong Wang, Jianwen Liang, Jianning Zhang, Yumin Qiu, Jun Shen, Xintao Shuai, * *Jun Tao,* * *and Wenhao Xia* *

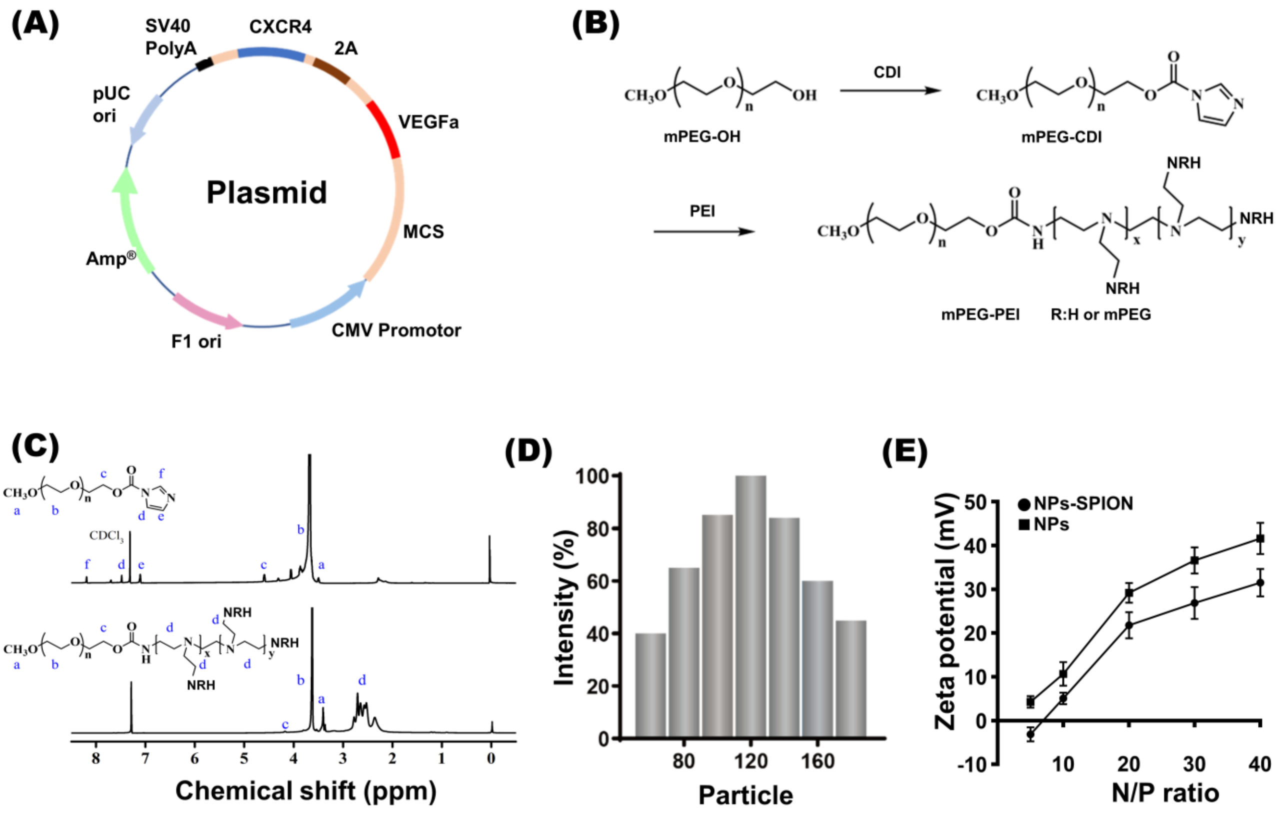

Figure S1. A) Illustration of the constructed plasmid equipped with VEGFa and CXCR4 genes together. B) Synthetic route of the copolymer mPEG-PEI and C) ^{1}H NMR spectra of mPEG-CDI and mPEG-PEI. D) Particle size distribution of nanoplex prepared at N/P 12 as measured by

dynamic light scattering (DLS). E) The zeta potentials of nanoplexes prepared at various N/P ratios. $n = 3$.

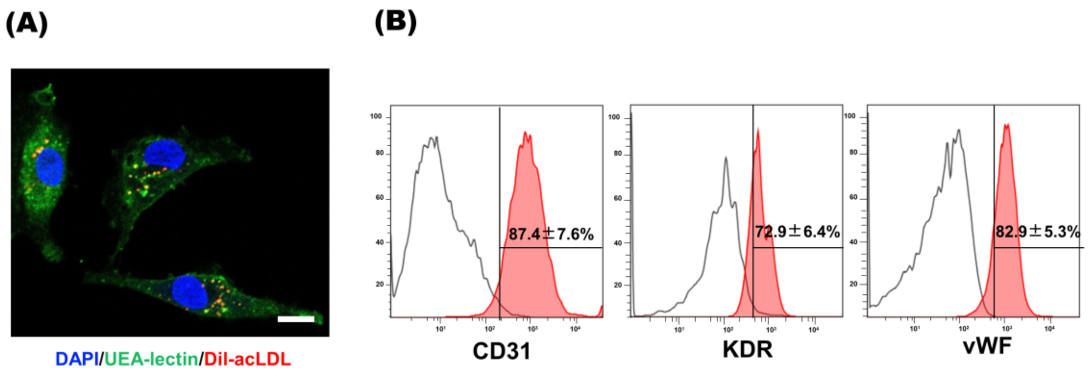

Figure S2. Phenotypic characterization of cultured EPCs. A) Representative photographs of cultured EPCs labeled with DAPI (blue), DiI-acLDL (red), FITC-lectin (green). Scale bar: 2 μm. B) Flow cytometric analysis of the endothelial markers CD31, vWF and KDR of cultured EPCs. Data are shown as mean ± SD, $n = 5$.

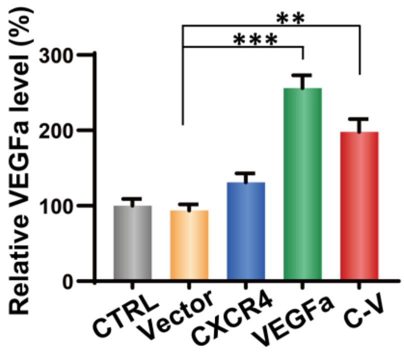

Figure S3. *In vitro* expressions of VEGFa in EPCs determined by ELISA. Data are shown as mean ± SD, $n = 5$. $^{**}P < 0.01$, $^{***}P < 0.001$. Abbreviations: CTRL, cells without treatment; Vecotr, EPCs transfected with Vector/NPs-SPION; CXCR4, EPCs transfected with CXCR4/NPs-SPION;

VEGFa, EPCs transfected with VEGFa/NPs-SPION; C-V, EPCs transfected with CXCR4-VEGFa/NPs-SPION; ELISA, Enzyme-Linked ImmunoSorbent Assay.

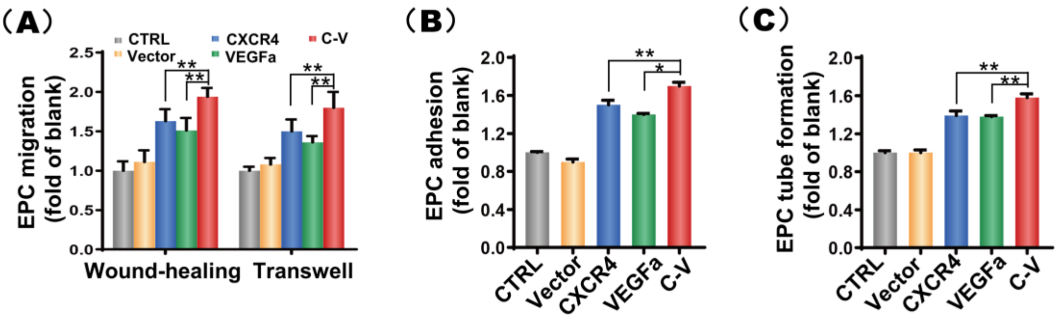

Figure S4. Quantitative analysis (Figure 4A) of *in vitro* function of EPCs enhanced by co-delivery of CXCR4 and VEGFa genes. A-C) Quantitative analysis of migration (A: Wound healing and Transwell), adhesion (B) and tube formation (C: Matrigel) of EPCs in different groups (5 groups: CTRL, Vector, CXCR4, VEGFa, C-V). Data are shown as mean ± SD, $n = 5$. $^*P < 0.05$, $^{**}P < 0.01$. Abbreviations: CTRL, cells without treatment; Vector, EPCs transfected with Vector/NPs-SPION; CXCR4, EPCs transfected with CXCR4/NPs-SPION; VEGFa, EPCs transfected with VEGFa/NPs-SPION; C-V, EPCs transfected with CXCR4-VEGFa/NPs-SPION.

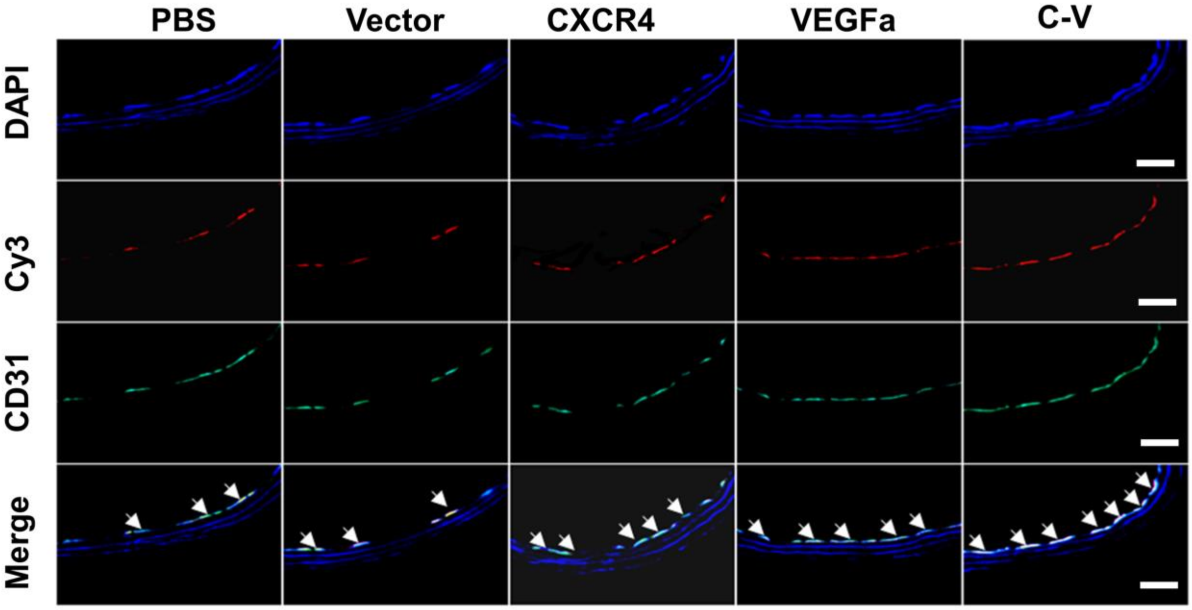

Figure S5. Representative photographs under fluorescent microscope showing Cy3-labeled EPCs (red) attached to CD31-stained endothelium (green) of injured carotid artery in nude rat receiving EPCs from different groups. Nuclei were stained with DAPI. Scale bar: 50 μm.

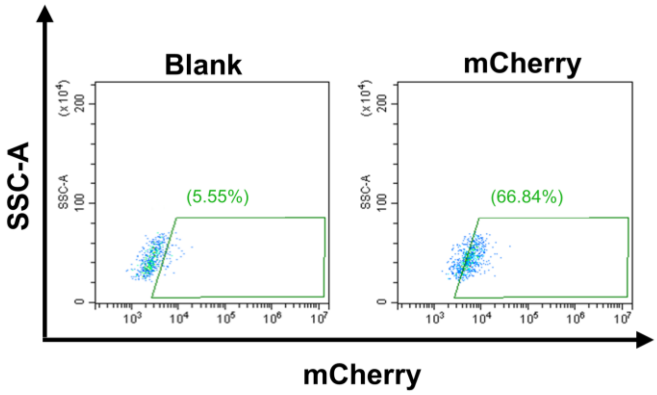

Figure S6. Flow cytometric analysis of mCherry expression in the infected EPCs.

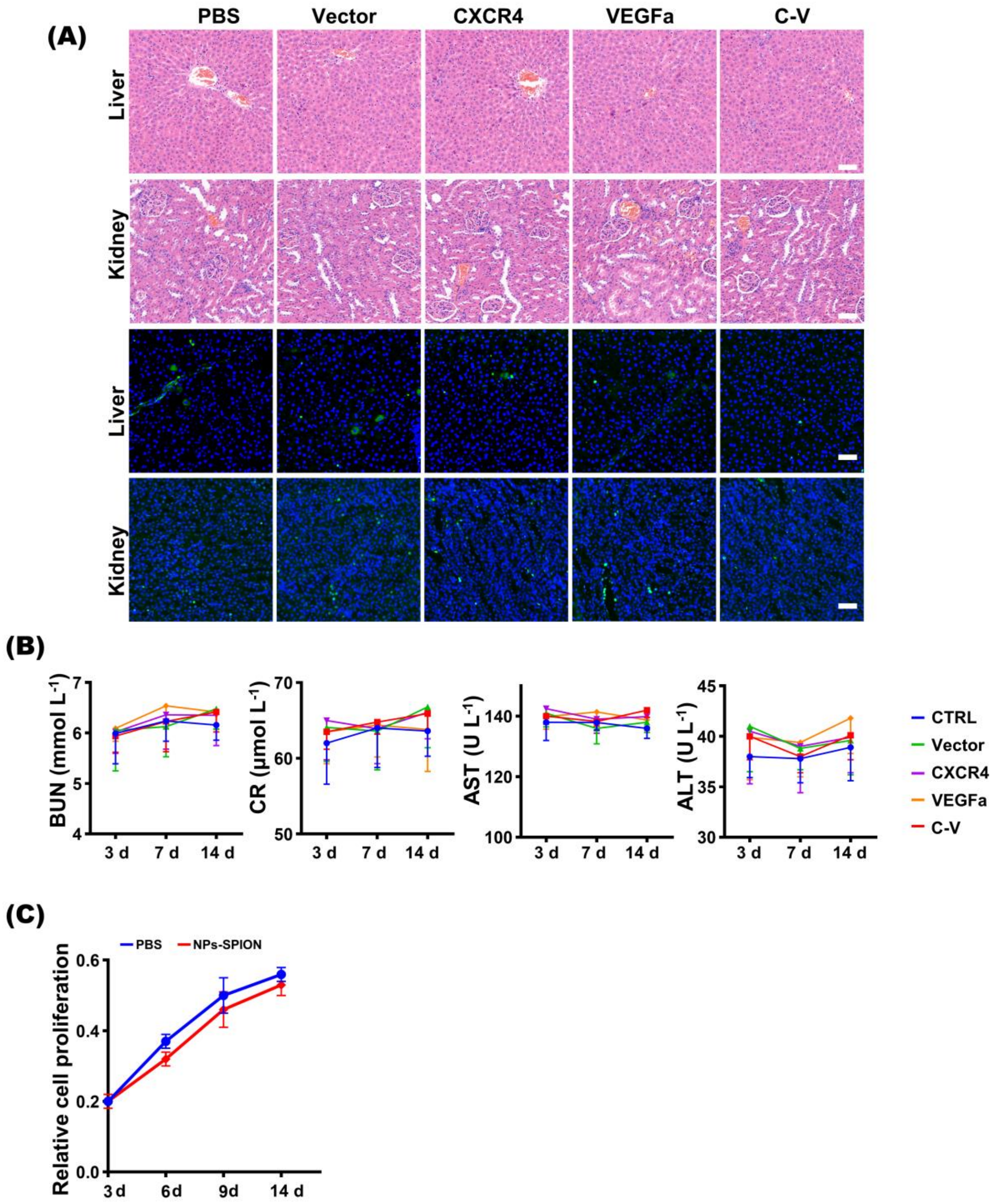

Figure S7. *In vivo* toxicological effect of the nanomedicine. A) H&E and TUNEL assay on liver and kidney tissues harvested at 14 d from different treatment groups. Scale bar: 100 μm. B) Serum levels of ALT, AST, CR, and BUN measured after EPCs transplantation (n = 5). C) Effect of SPION on the lifespan of differentiated endothelial cells assessed by CCK-8 assay. Abbreviations: PBS, treatment with the same volume of PBS; Vector, EPCs transfected with Vector/NPs-SPION;

CXCR4, EPCs transfected with CXCR4/NPs-SPION; VEGFa, EPCs transfected with VEGFa/NPs-SPION; C-V, EPCs transfected with CXCR4-VEGFa/NPs-SPION; ALT, alanine transaminase; AST, aspartate transaminase; CR, creatinine; BUN, blood urea nitrogen; H&E, hematoxylin-eosin staining; TUNEL, TdT-mediated dUTP Nick-End Labeling; NPs-SPION, nanocarrier PEG-PEI-SPION; D, day.

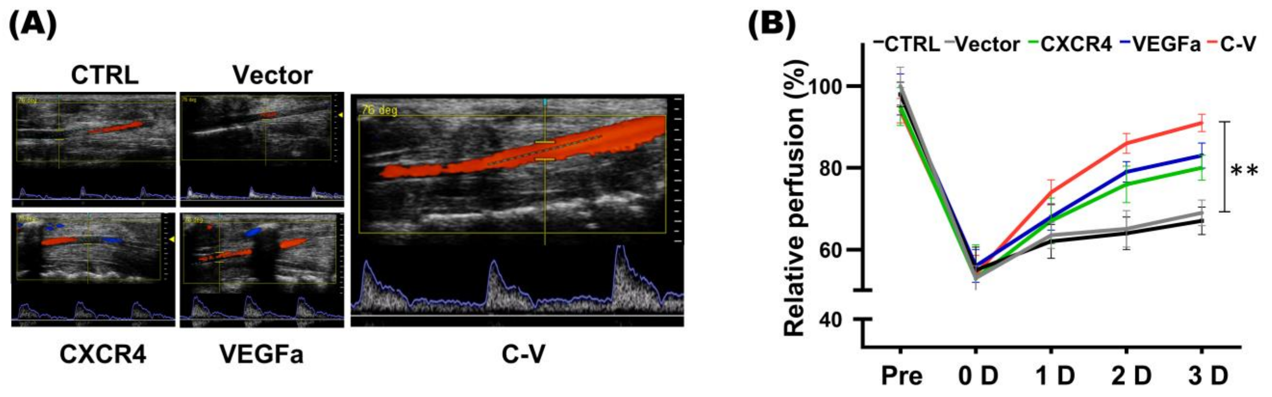

Figure S8. Blood perfusion in different groups were monitored with the laser Doppler flowmetry. A) Representative photographs of rat carotid blood flow in different groups conducted. B) The statistical results showed that the mean blood flow was significantly improved in C-V group at 3 d after EPCs transplantation. Data are shown as mean ± SD, n = 5. ***P* < 0.01. Abbreviations: PBS, treatment with the same volume of PBS; Vector, EPCs transfected with Vector/NPs-SPION; CXCR4, EPCs transfected with CXCR4/NPs-SPION; VEGFa, EPCs transfected with VEGFa/NPs-SPION; C-V, EPCs transfected with CXCR4-VEGFa/NPs-SPION; D, day.

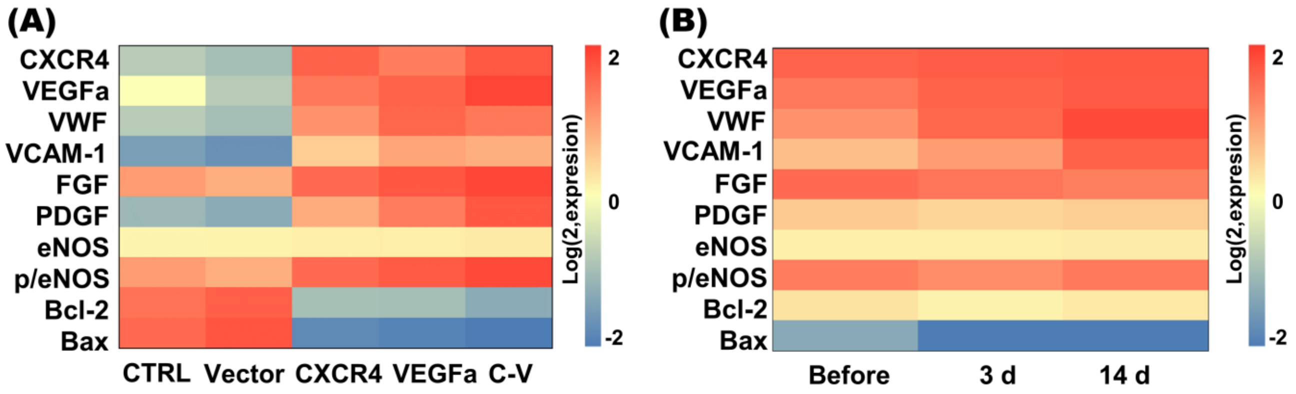

Figure S9. The mRNA levels of endothelialization-related genes and apoptosis-related genes of the host endothelial cells and injected engineered EPCs. The mRNA levels of endothelialization-related biological molecules (CXCR4, VEGFa, VWF, VCAM-1, FGF, PDGF and eNOS) and apoptosis-related genes (Bcl-2 and Bax) of host endothelial cells at 3 d after EPCs transplantation (A) and of engineered EPCs in C-V group before and after injected at 3 d and 14 d (B) were assessed by qPCR. *n* = 5. Abbreviations: VWF, von Willebrand factor; VCAM-1, Vascular cell adhesion protein 1; FGF, fibroblast growth factor; PDGF, platelet derived growth factor; eNOS: endothelial nitric oxide synthase; Bcl-2, B-cell lymphoma 2; Bax, bcl-2-like protein 4; PBS, treatment with the same volume of PBS; Vector, EPCs transfected with Vector/NPs-SPION; CXCR4, EPCs transfected with CXCR4/NPs-SPION; VEGFa, EPCs transfected with VEGFa/NPs-SPION; C-V, EPCs transfected with CXCR4-VEGFa/NPs-SPION; D, day.
